# Supplementary material for: Identification of Virulence Genes Associated with Pathogenicity of Translocating Escherichia coli with Special Reference to the Type 6 Secretion System
Source: Microorganisms. 2024 Sep 6;12(9):1851. doi: 10.3390/microorganisms12091851 (PMC11433802; doi:10.3390/microorganisms12091851)
Supplement: Supplementary file 1 [file microorganisms-12-01851-s001.zip › microorganisms-3190044-supplementary.pdf]

Supplementary Table S1: Primer sequences and PCR protocols used for detection of *E. coli* virulence genes (VGs) associated with adhesive-invasive *E. coli* (AIEC) and type 6 secretion system (T6SS).

**Gene**	Denaturation and temp.	Annealing temp.	Extension	No. of cycles	Primer Sequences 5' → 3'	Amplicon size (bp)	Ref. no.
*htr*A	95ºC 1 min	55ºC 1 min	72ºC 2 min	29	F- TTCCAGCAGTTCTTCGGTGA<br>R- ATCAGTTCGCCGTTCAGGTT	530	(57)
*omp*C	95ºC 30s	53ºC 1 min	72ºC 2 min	29	F- GCGCCGACATCAACGTATTT<br>R- GCCAACAAAGCGCAGAACTT	141	
*lpf*A	95ºC 1 min	65ºC 1 min	72ºC 1 min	29	F- AGGCGGTGCATTCACTCTGGCATCT<br>R- CCGCGTCGATAGCGGTATAGGCAGA	448	
*dsb*A	95ºC 30s	56ºC 1 min	72ºC 2 min	29	F- CTGCCGGAAGGCGTGAAC<br>R- GCTGTTCCACGCCGCGTC	237	
*afa*C	94ºC 1 min	65ºC 1 min	72ºC 2 min	29	F- CGGCTTTTCTGCTGAACTGGCAGGC<br>R- CCGTCAGCCCCCACGGCAGACC	627	
*clb*A	95ºC 30s	60ºC 1 min	72ºC 2 min	29	F- CTAGATTATCCGTGGCGATTC<br>R- CAGATACACAGATACCATTCA	872	(58)
*hly*A	94ºC 30s	65ºC 30s	68ºC 75s	25	F- AACAAGGATAAGCACTGTTCTGGCT<br>R- ACCATATAAGCGGTCATTCCCGTCA	1177	(34)
*iut*A	94ºC 1 min	55ºC 1 min	72ºC 1 min	30	F- GGCTGGACATCATGGGAACTGG<br>R- CGTCGGGAACGGGTAGAATCG	300	
*iro*N	94ºC 30s	55ºC 30s	72ºC 45s	30	F- AAGTCAAAGCAGGGGTTGCCCG<br>R- GACGCCGACATTAAGACGCAG	665	
*fyu*A	94ºC 30s	57ºC 30s	72ºC 1 min	30	F- TGATTAACCCCCGCGACGGGAA<br>R- CGCAGTAGGCACGATGTTGTA	780	
*ire*A	95ºC 1 min	50ºC 30s	72ºC 1 min	30	F- GATGACTCAGCCACGGGTAA<br>R- CCAGGACTCACCTCACGAAT	254	
IbeA	94ºC 30s	55ºC 30s	72ºC 90s	35	F- TGGAACCCGCTCGTAATATAC<br>R- CTGCCTGTTCAAGCATTGCA	900	
*vgr*G	94ºC 30s	55ºC 30s	72ºC 1 min	35	F- TCACAGGACGAAGCCCGTTAC<br>R- CGTCGCGGTCAAAGTCGAGAT	958	(37)
*clp*V	94ºC 30s	55ºC 30s	72ºC 90s	35	F- GCACGTCAGCCACAGGTCAC<br>R- ACGGTATGCGGTTCCTGGTA	1487	
*arcA**	94ºC 30s	55ºC 30s	72ºC 90s	35	F- TGTGAAAACCCAGGCAAAA<br>R- TTCCAGATCACCGCAGAAG	195	(21)
*gapA**	94ºC 30s	55ºC 30s	72ºC 90s	35	F- CGAAGTTGGTGTTGACGTTG<br>R- TTGTCGAAGTTAGCGCCTTT	156	(21)

* Housekeeping genes *arc*A (aerobic respiration control A) and *gap*A (Glyceraldehyde-3- phosphate dehydrogenase).

Supplementary Table S2

Identification of type 6 secretion system (T6SS) virulence genes (VGs) from common (C) types and single (S) types representing strains from adult community-acquired (CA) UTI, child hospital-acquired (HA) UTI, adult septicaemia, adult HA-UTI, child diarrhoea and adult inflammatory bowel disease (IBD).

C-types	*clp*V	*vgr*G	No. of isolates represented	Total	Source
C1	+	+	20	67/75 (89%)	Adult CA-UTI
C2	+	+	5		
C3			4		
C4	+	+	10		
C5			2		
C6	+	+	11		
C7	+	+	6		
C8			2		
C9	+	+	11		
C10	+	+	2		
C11	+	+	2		
C12	+	+	4	62/75 (83%)	Child HA-UTI
C13	+	+	4		
C14	+	+	3		
C15	+		3		
C16	+	+	5		
C17		+	2		
C18	+	+	5		
C19			2		
C20			6		
C21			3		
C22	+	+	30		
C23	+	+	3		
C24			2		
C25	+	+	3		
C26	+	+	5	49/74 (66%)	Adult septicaemia
C27			9		
C28	+	+	7		
C29	+	+	2		
C30	+	+	5		
C31		+	2		
C32	+	+	8		
C33	+	+	3		
C34	+	+	6		
C35	+	+	4		
C36	+	+	7		
C37			16		
S1-S30	+	+	30	3/30 (10%)	Child diarrhoea
S31-S70	+	+	40	17/40 (43%)	Adult HA-UTI
S71-S95	+	+	25	8/25 (32%)	Adult IBD
